# Improving Photovoltaic Performance of Hybrid Organic-Inorganic MAGeI_3_ Perovskite Solar Cells via Numerical Optimization of Carrier Transport Materials (HTLs/ETLs)

**DOI:** 10.3390/nano13152221

**Published:** 2023-07-31

**Authors:** Tariq AlZoubi, Bachar Mourched, Mahmoud Al Gharram, Ghaseb Makhadmeh, Osama Abu Noqta

**Affiliations:** 1 College of Engineering and Technology, American University of the Middle East, Egaila 54200, Kuwait; 2 Department of Physics, School of Basic Sciences and Humanities, German Jordanian University, Amman 11180, Jordan; 3 Bio-Medical Physics Laboratory, Department of Physics, Jordan University of Science and Technology, Irbid 22110, Jordan; 4 MEU Research Unit, Middle East University, Amman 11831, Jordan

**Keywords:** numerical modeling, perovskites, MAGeI_3_, solar cell, SCAPS-1D

## Abstract

In this study, a hybrid organic–inorganic perovskite solar cell (PSC) based on methylammonium germanium triiodide (MAGeI_3_), which is composed of methylammonium ($CH_{3}NH_{3}^{+})$ cations and germanium triiodide ($GeI_{3}^{-}$) anions, has been numerically studied using SCAPS-1d codes. An extensive investigation of various electron transport layers (ETLs) and hole transport layers (HTLs) was conducted to identify the most optimal device configuration. The FTO/ZnOS/MAGeI_3_/PEDOT-WO_3_ structure performed the highest efficiency of all combinations tested, with an impressive optimized efficiency of 15.84%. This configuration exhibited a *V_oc_* of 1.38 V, J_sc_ of 13.79 mA/cm^2^, and FF of 82.58%. J-V characteristics and external quantum efficiency (EQE) measurements indicate that this device offers superior performance, as it has reduced current leakage, improved electron and hole extraction characteristics, and reduced trap-assisted interfacial recombination. Optimum device performance was achieved at active layer thickness of 560 nm. These findings may also serve as a basis for developing lightweight and ultra-thin solar cells, in addition to improving overall efficiency. Furthermore, a comprehensive correlation study was conducted to evaluate the optimum thickness and doping level for both ZnOS-ETL and PEDOT-WO_3_-HTL. The photovoltaic performance parameters of the FTO/ZnOS/MAGeI_3_/PEDOT-WO_3_ structure were analyzed over a wide temperature range (275 K to 450 K). The structure exhibited stable performance at elevated operating temperatures up to 385 K, with only minimal degradation in PCE of approximately 0.42%. Our study underscores the promise of utilizing cost-effective and long-term stability materials like ZnOS and PEDOT-WO_3_ alongside the toxic-free MAGeI_3_ perovskite. This combination exhibits significant potential for eco-friendly PSC, paving the way for the development of highly efficient ultra-thin PSC.

## 1. Introduction

Recent progress and development of hybrid organic–inorganic perovskites solar cells has made a significant contribution to the field of photovoltaics. Their efficiency has increased dramatically from 4% to 26% in a relatively short amount of time [1,2,3,4]. In comparison, other technologies, such as Si PV technology, have taken a significantly longer time to achieve the same level of improvement [4,5,6,7,8]. As a result of this rapid and significant development, hybrid perovskite solar cells are expected to have a profound impact on the future of solar energy. Hybrid lead-based perovskites are based on the MAPbX3 formula, where MA is an organic or inorganic cation and X is a halide. These compounds exhibit exceptional optoelectronic properties, such as high optical absorption coefficients, low trap density, and shallow defect states [9,10,11,12,13,14]. In addition, these materials are highly promising for the future advancement of photovoltaics and solar energy as they can be processed from solutions at or near room temperature, as opposed to silicon solar panels, which require significantly higher deposition temperatures [15]. Several potential alternatives to conventional perovskites have emerged in recent years, including CH3NH3PbCl3, because of its exceptional properties, including high UV and visible absorption, adjustable band gaps, and long electron-hole diffusion lengths [16]. Metal halide perovskites are ideal candidates for the next advancement in PV technology for their ability to be processed from solutions and their low cost. Their durability, however, is affected by environmental factors, such as heat, moisture, and oxygen, as well as the use of lead, which is toxic. There are, however, numerous researcher in the field of photovoltaic technology who have recommended Bi, Ge, Sb, In, Ag, Te, or Sn as potential replacement elements with similar chemical and physical properties to lead [17,18]. The toxic-free nature of Ge-based perovskite solar cells, such as CH3NH3GeI_3_, has recently made them prospective candidates for the active layer in current PV devices technology. Therefore, Germanium-based perovskites present a viable alternative to traditional lead-based solar cells. A hybrid organic–inorganic germanium perovskite, MAGeI_3_, is a perovskite with adjustable bandgap that is easily synthesized by solution processing [19]. Additionally, MAGeI_3_ has demonstrated superior optical and electronic properties, improved electron and hole behavior, and superior hole behavior compared to other perovskite materials [20,21]. A further advantage of Ge is its ability to maintain stability even at elevated temperatures up to 150 °C, making it an ideal choice for many PSC applications [12]. As a result of these properties, germanium-based perovskite solar cells may prove to be both more efficient and more environmentally friendly in the future. As a result of the use of methylammonium germanium iodine perovskites (CH3NH3GeI3) as an active layer, Deepthi et al. achieved a remarkable power conversion efficiency (PCE) of 12.98% [22,23]. The result was achieved by combining a variety of ETL and HTL materials, along with powerful modeling tools such as SCAPS 1D.

The use of numerical simulations and modeling is essential for the advancement and optimization of solar cell technology. Using simulations to predict solar cell performance has many benefits, including optimizing design, reducing experimentation requirements, and improving understanding of physical processes [24]. Additionally, simulations are useful tools for assessing materials parameters and the potential for new materials. The accuracy of a simulation depends on the calibration of physical model parameters. The Solar Cell Capacitance Simulator (SCAPS-1D) offers a unique advantage by providing simulations under both light and dark conditions based on solving the Poisson and continuity equations, incorporating recombination mechanisms, batch calculations, and defect-level analysis [25,26]. The key to enhancing the efficiency of PSC (Perovskite Solar Cell) devices lies in the careful and strategic selection of both the absorber and carrier transport layers, particularly the electron transport layer (ETL) and hole transport layer (HTL), along with their respective thicknesses. It is crucial to find the most optimal combination of ETLs and HTLs to achieve significant improvements in device efficiency. Thus, ETLs and HTLs should be combined optimally for device efficiency improvement [27]. This can be accomplished by taking into account several parameters, such as the type of organic–inorganic materials used, their thickness, optical and electrical parameters, and their interfacial properties. PV performance is dependent on the exact combination of material parameters for each device configuration. Therefore, the different combinations of materials should be tested thoroughly in accordance with the desired properties.

Hybrid organic–inorganic perovskite solar cells require Hole Transport Layers (HTLs) in order to move holes efficiently from the active layer to the electrodes. The performance of HTL materials, particularly their electrical conductivity and hole mobility, significantly impacts the overall efficiency of these solar cells. Extensive research has revealed that optimizing HTL can lead to substantial improvements in perovskite solar cell efficiency by minimizing charge recombination and enhancing charge collection [28]. In fact, specific materials have been identified to exhibit exceptional Power Conversion Efficiency (PCE) when employed as HTL in perovskite solar cell devices. These findings highlight the crucial role of HTLs in advancing perovskite solar cells’ performance and underscore the importance of selecting suitable materials for this purpose. It has been reported that certain materials have demonstrated extraordinary PCE when used as the HTL in PSC devices [17,18]. Other studies achieved an extraordinary PCE using materials like D-PBTTT-14 and PEDOT: PSS as HTL in the PSC device. For instance, PEDOT-WO_3_ is a combination of conductive polymer Poly(3,4-ethylenedioxythiophene) (PEDOT) and transition metal oxide tungsten trioxide (WO_3_) that has been widely used as a hole transport layer in organic photovoltaic cells. Known for its high electrical conductivity, stability, and compatibility with various organic materials, PEDOT-WO_3_ can enhance the performance of organic cells by decreasing charge recombination, improving charge transport, and limiting charge trap formation [18,28,29]. As a result, PEDOT-WO_3_ has demonstrated potential as a high-performance HTL in OPV cells and is expected to continue gaining popularity among researchers as new candidate for optimizing its properties are developed.

Efficient functioning of a solar cell depends a lot on the ability of electron transport layer (ETL) material to collect and move electrons to the electrodes. To ensure optimum performance of the cell, it is important that the ETL material remains stable under diverse operating conditions, including but not limited to high temperature, humidity, and light exposure. It should have low recombination rates to minimize the loss of carriers and to improve the efficiency of the cell and must match the absorption spectrum of the solar cell to improve light absorption and higher efficiency. In summary, the ETL material plays a crucial role in the overall efficiency of a solar cell. For instance, in lead-based perovskite solar cells, TiO_2_ was employed as an ETL, leading to a PCE of 17.46%. Another study showed an improvement in PCE to 17.08% by using SnO_2_ as an ETL material. The use of TiO_2_ as an ETL is, however, limited due to its low carrier mobility, high deposition temperature, and negative effects on device stability under UV light [30,31,32]. Therefore, its choice has a significant impact on the performance of the cell. As a consequence of their high costs and chemical instability, it is evident there is a high demand for cost-effective and more stable alternatives to high-temperature processed ETLs and HTLs.

This study investigates the impact of different transport layers, particularly PEDOT-WO_3_, on lead-free MAGeI_3_-based PSC devices under solar illumination at AM1.5. Our study focused on investigating a lead-free MAGeI_3_ perovskite solar cell (PSC) device structure, with the primary objective of identifying the ideal combination of carrier transport materials. We specifically explored various configurations of ETLs and HTLs, focusing on a hybrid organic–inorganic perovskite solar cell as the active material. PEDOT-WO_3_ demonstrates good stability and compatibility with the perovskite layer, enabling long-term device operation and enhanced device durability. It has high stability against moisture, low material cost, which can be deposited at low temperature [11,33,34]. Additionally, the absorber thickness of MAGeI_3_ is optimized to attain maximum efficiency in the proposed PSC device structure. The improvement of photovoltaic parameters in lead-free PSC devices is a sign of effective carrier transport layers with a noticeable EQE parameter for the simulated PSC. The structure of the paper is outlined as follows: the simulation parameters for PSC device structures are presented in Section 2, while the mathematical modeling and its results are discussed in Section 3.

## 2. Device Structure and Simulation Methodology

**Figure 1:**
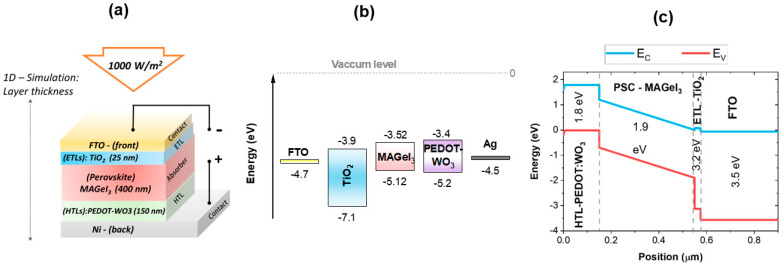
Schematic diagram of the proposed lead-free PCS based on the FTO/TiO_2_/PEDOT-WO_3_/Ag structure. (**a**) Illustrates the layer scheme. (**b**) Depicts the band alignments. (**c**) Shows the variations in conduction and valence bands, along with the energy gap, as a function of position.

This study was conducted using the SCAPS-1D software package developed by researchers at the Department of Electronics and Information Systems (ELIS) at the University of Gent in Belgium [25]. Solar cells characterized by perovskites, CZTS, Si, CdTe, and CIGS have been successfully simulated with SCAPS-1D to determine their electrical and optical characteristics, performance, and spectral response. In fact, one of the major advantages of SCAPS is that the simulation results are in good agreement with the results of the corresponding experiments, such as those for perovskite, CZTS, Si, CdTe, and CIGS solar cells [34,35,36,37,38]. In this advanced numerical simulation tool, Poisson’s equation and the continuity equation are effectively solved, which allows us to accurately simulate the behavior of free electrons and holes in the conduction and valence bands. As part of its capabilities, it is possible to compute and observe various electrical properties and parameters crucial to the evaluation of solar cell performance, such as the current density–voltage curve (J-V curve), the energy band structure of the heterojunction, quantum efficiency (QE), open circuit voltages (*V_oc_*), short circuit currents (J_SC_), current density, power conversion efficiency (PCE), and fill factor (FF), among others. All simulated PSC structures were subjected to standard test conditions (STC), including a temperature of 300 K, an intensity of 1000 W/m^2^, and an air mass of AM1.5 G. The absorber layer (active layer MAGeI_3_) was positioned between the hole transport layer (HTL) and electron transport layer (ETL), enabling efficient energy conversion as shown in Figure 1.

The main objective of this study is to determine the optimal performance of a lead-free PSC using MAGeI_3_ as the active layer by employing an n-i-p structure and a variety of ETLs and HTLs. Based on SCAPS-1D algorithms and codes, the photovoltaic performance parameters of the lead-free MAGeI_3_ PSC were evaluated. As part of the evaluation process, the ETL and HTL materials were changed at different absorber thicknesses, bias voltages, and doping concentrations. As a result of this simulation study, a lead-free MAGeI_3_ PSC was designed and optimized using the parameters determined during the simulation study. For the reference model (FTO/TiO_2_/MAGeI_3_/PEDOT-WO_3_), the absorber layer thickness was initially set at 400 nm (as shown in Figure 1).

As shown in Table 1, all the basic physical parameters that have been incorporated into the simulation study of the proposed structure of MAGeI_3_ have been presented [12,32,33,34,35]. PSC devices achieve optimum performance when the interfacial properties and contacts of the devices are of the highest quality. Table 2 summarizes the parameters used in the model to account for interface defects at perovskite HTL/ MAGeI_3_/ETL interfaces. This allows for an accurate simulation of the actual device behavior and thus allows for the optimization of device parameters. Moreover, the simulation also provides a better understanding of the underlying physical mechanism of charge transport and recombination, which is critical for the optimization of PV device performance.

Two primary numerical studies were executed to enhance the performance of the MAGeI_3_-based PSC structure. The first study involved testing and comparing three alternative Electron Transport Layer (ETL) materials with the initial reference structure’s TiO_2_ (Titanium Dioxide) ETL. The evaluated materials included Tin Dioxide (SnO_2_), PC61 Phenyl-C61-Butyric Acid Methyl Ester (PC61PM), and Zinc Oxide Sulfide (ZnOS). Table 3 provides a summary of the parameters associated with these three novel ETL materials utilized in this study. However, the second numerical study involved testing and comparing three Hole Transport Layer (HTL) materials: Nickel Oxide (NiO), Spiro-OMeTAD (Spiro-2,2′,7,7′-tetrakis(N,N-di-p-methoxyphenylamine)9,9′-spirobifluorene), and PEDOT (Poly(3,4-ethylenedioxythiophene)), with the HTL of the initial reference structure, PEDOT-WO_3_ (Poly(3,4-ethylenedioxythiophene):tungsten trioxide). Table 4 provides a comprehensive list of parameters associated with these four HTL materials [16,28].

The primary objectives of these studies are to improve the power conversion efficiency (PCE), replace the toxic absorber MAPbI_3_ with lead-free absorber (MAGeI_3_), and enhance the overall stability of the device. The reference model in this study utilizes TiO_2_-ETL because it is the most commonly used ETL material in perovskite solar cells (PSCs) technology and has shown promising results in terms of PCE. For instance, Himaa et al. reported a notable advancement in solar cell technology through the integration of TiO_2_/Perovskites and spiro-OMeTAD layers in an organic and inorganic perovskite solar cell, revealing maximum power conversion efficiencies of 18.16% for CH_3_NH_3_PbI_3_ and 9.56% for CH_3_NH_3_SnI_3_ [39]. Metal oxides, including TiO_2_ and SnO_2_, have received significant attention as electron transport layers (ETLs) in high-performance PSCs. Among these, TiO_2_ stands out as the most frequently used ETL in PSCs. However, to achieve optimal crystallinity and conductivity, TiO_2_ must be annealed at high temperatures, typically at 450 °C [40,41]. Likewise, SnO_2_ also requires post-annealing at temperatures exceeding 180 °C [42]. This high annealing temperature step adds complexity and cost to the device manufacturing process.

In this work, extensive simulations and analyses have been conducted to address these challenges by modifying the properties of various layers of the ETL, including TiO_2_, SnO_2_, PC61PM, and ZnOS. The high demand for cost-effective and chemically stable alternatives to conventional high-temperature processed ETLs and HTLs is evident due to their high costs and inherent chemical instability. In this context, PEDOT/WO_3_ has recently emerged as a focus in many experimental studies due to its exceptional properties and excellent long-term stability, which have been observed to have a direct impact during the injection/ejection process of the charge carrier into PEDOT/WO_3_ stacked structures. The first successful use of WO_3_ as a hole transfer layer for organic solar cells (OSCs) was reported by Stubhan et al. in 2012 [43]. This finding revealed that WO_3_ could effectively replace PEDOT: PSS, a commonly used material for this purpose. The research was further enhanced in 2018 by Zheng et al., who reported the development of an organic solar cell based on a water-based emulsion of PEDOT: PSS combined with WO_3_. The device demonstrated enhanced performance and prolonged carrier lifetime, which makes it a valuable advancement in PCSs [39]. As a result, the open-circuit voltage was increased and power conversion efficiency was improved. The combination of WO_x_:PEDOT:PSS increased device stability and temperature operation as well. Therefore, PEDOT-WO_3_ HTL is an attractive option for efficient and stable PCSs due to its excellent transparency, superior electrical properties, and environmental stability. Overall, these studies collectively contribute to the advancement of perovskite solar cell technology by exploring novel ETLs and HTLs alternatives with improved efficiency, stability, and cost-effectiveness.

## 3. Results and Discussion

Charge transporting layers (CTLs) of perovskite solar cells, including ETLs and HTLs, are crucial to their performance. Indeed, CTLs facilitate the injection of electrons or holes into electrodes by efficiently extracting electrons or holes. Thus, it is imperative to carefully select ETL/HTL materials to obtain the best performance from PSC structures. CTLs should have superior electrical conductivity, high mobility, large band gap, and high thermal stability to ensure the overall PSC stability and high performance. They should also possess a low work function and suitable energy level for efficient charge transport. Moreover, CTLs should be compatible with perovskite materials to maximize device performance. The CTLs should also be capable of forming an interfacial layer with the perovskite as well as forming a homogeneous film with a minimum number of defects or traps. This can help to reduce charge recombination and increase the device lifetime and efficiency. Another important feature of CTLs is their ability to block oxygen and moisture, preventing degradation of the device and preserving its optimal performance. The perovskite HTL/MAGeI_3_/ETL interfaces offer several advantages. The improved charge transport properties of these devices result in a reduction of recombination losses and an improvement in efficiency. In addition, controlling these interfaces has demonstrated enhanced resistance to environmental factors, such as humidity, light exposure, and temperature fluctuations, resulting in longer device lifetimes [44]. Further, they are designed to achieve optimal energy level alignment, an important factor in determining charge extraction and injection efficiency. Considering these benefits, the perovskite HTL/MAGeI_3_/ETL interfaces are a promising configuration for advanced and efficient lead-free MAGeI_3_ PV devices. The energy level alignment between the absorber (perovskite) and the electron transport layer ETL materials is a key factor highlighted in numerous studies [45,46]. This alignment is quantified by the conducting band offset (CBO), which represents the difference in electron affinity between the ETL/absorber or absorber/HTL. Thus, precise engineering and control of the interface between the ETL/HTL layers and perovskite are crucial for addressing the CBO and achieving high-efficiency planar perovskite solar cells [46]. One of the major challenges in these solar cells is recombination loss occurring across the interfaces, particularly at the ETL/absorber interface, which can result in voltage reduction [47]. Tackling this issue is essential for further optimizing the performance of planar perovskite solar cells. In this section, we present a comprehensive modeling and analysis of the influence of HTL and ETL materials on the performance of MAGeI_3_-based perovskite solar cells. During this investigation, we aim to gain a better understanding of the relationship between these materials and the efficiency of solar cells.

### 3.1. Effect of ETL Materials on PSC Photovoltaics Performance Parameters

**Figure 2:**
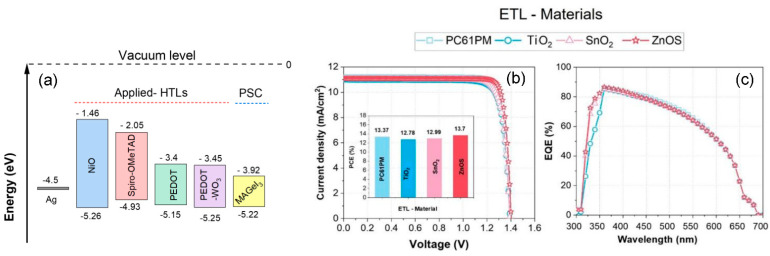
Impact of the ETL materials on PSC performance: (**a**) ETLs bands alignment with respect to absorber (MAGeI_3_) layer. (**b**) J-V characteristics; the histograms in the inset depict the maximum Power Conversion Efficiency (PCE) of PSC as a function of ETL materials. (**c**) External Quantum efficiency (EQE) for the simulated devices with different ETL materials.

A total of four different ETL materials were examined and compared to the reference structure’s ETL (TiO_2_). Table 3 presents a comprehensive overview of the electrical properties associated with these ETL materials. Figure 2b illustrates the current–voltage (J-V) characteristics of the device with respect to the ETL materials used in the reference sample.

With a short-circuit current density of 11.2 mA/cm^2^ and a V_OC_ of 1.42 V, the ZnOS ETL material emerged as the top performer with PCE of 13.7%. This outstanding performance can be attributed to ZnOS’s favorable conduction band offset (CBO), which makes it an ideal material for use as an ETL.

Figure 2b inset presents a histogram illustrating the maximum Power Conversion Efficiency (PCE), showcasing that the structure with ZnOS as the ETL material exhibited the highest PCE compared to the other ETL materials. It is, however, essential to consider the impact of the different band alignments observed for various ETL materials, as shown in Figure 2a. Due to these variations in band alignments, the interface quality changes and subsequently affects the density of trap defect states. The defect density at the ETL–perovskite interface is closely correlated with the energy level alignment, which is referred to as the conducting band offset (CBO). The CBO represents the difference in electron affinity between the absorber material (MAGeI_3_) and ETL material. In order to achieve high-efficiency planar PSCs, proper interface engineering and control are vital at the ETL/absorber junction. A negative CBO indicates the formation of a spike-like structure at the ETL/absorber layer interface. This spike acts as a barrier for photo-generated electrons, preventing them from reaching the ETL/absorber interface. As a result, this barrier enhances photogeneration of free charge carriers and suppresses recombination rates at the interface, thereby reducing the *V_oc_* as shown in Figure 2b. With this spike structure, solar cells exhibit greater power conversion efficiency, as demonstrated with ZnOS and TiO_2_ as ETL materials that have positive CBO values of −0.32 eV and −0.02 eV, respectively. The high PCE observed with ZnOS as the ETL is due to its strong band bending capability as well as its ability to create a good interface potential for charge carrier separation. In contrast, a positive CBO indicates that the ETL’s CBO level is lower than that of the perovskite, leading to the formation of an energy cliff at the ETL/perovskite interface. This negative CBO was observed for SnO_2_ and PC61PM ETLs with CBO values of 0.08 eV. As a result, ETLs with a smaller built-in potential barrier result in less band bending and increased interface recombination, leading to a significant decrease in PCE, as observed for SnO_2_ and PC61PM ETLs. Additionally, ZnOS exhibits the highest electron mobility among the ETL materials studied, enabling faster extraction of photo-generated charge carriers to both electrodes. According to Figure 2c, the EQE improved even further as a result of enhanced electron mobility. Therefore, by establishing selective electron contact with the perovskite layer, the ZnOS ETL enhances the efficiency of electron extraction from the perovskite active layer while blocking holes. The effective electron extraction reduces carrier recombination, enhances carrier separation, and ultimately improves the performance of the device. For an ideal ETL, it is necessary to select an n-type semiconductor that exhibits a wide bandgap energy and is transparent to visible light. It should also demonstrate higher carrier mobility and electrical conductivity to ensure effective electron transportation at the front contact of the device. Remarkably, these criteria align perfectly with the characteristics of the ZnOS ETL, as depicted in Figure 2 and Table 3. The relationship between incident light wavelength and external quantum efficiency (EQE) is shown in Figure 2c. Quantum efficiency refers to the percentage of carriers that are collected from incident photons of a particular wavelength or energy. Notably, an enhancement in EQE from 69% to 83% was observed specifically at a wavelength of 350 nm, precisely coinciding with ZnOS bandgap energy. Due to its higher transparency at 350 nm, ZnOS captures more light at this wavelength than other ETLs. Consequently, more carriers are created and collected in the external circuit, resulting in a significant increase in the overall EQE.

**Figure 3:**
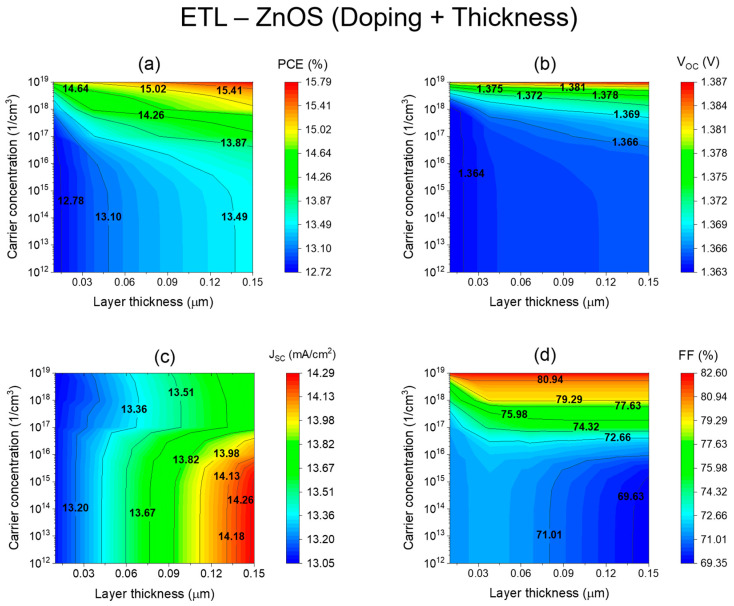
Impact of the ETL (ZNOS) thickness and carrier concentration on key photovoltaic parameters: (**a**) Power Conversion Efficiency (PCE). (**b**) Open-Circuit Voltage (*V_oc_).* (**c**) Short-Circuit Current Density (Jsc). (**d**) Fill Factor (FF).

**Figure 4:**
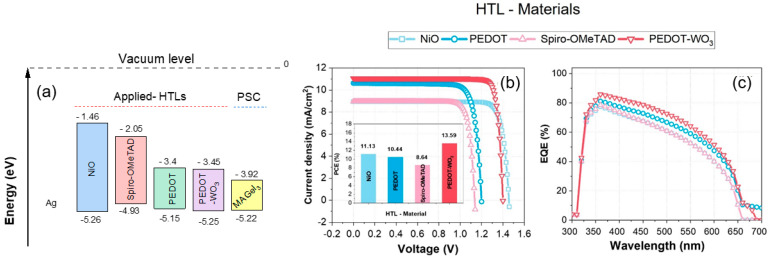
Impact of the HTL materials on PSC performance: (**a**) HTLs bands alignment with respect to absorber (MAGeI_3_) layer. (**b**) J-V characteristics; the histograms in the inset depict the maximum Power Conversion Efficiency (PCE) of PSC as a function of HTL materials. (**c**) External Quantum efficiency (EQE) for the simulated devices with different HTL materials.

**Figure 5:**
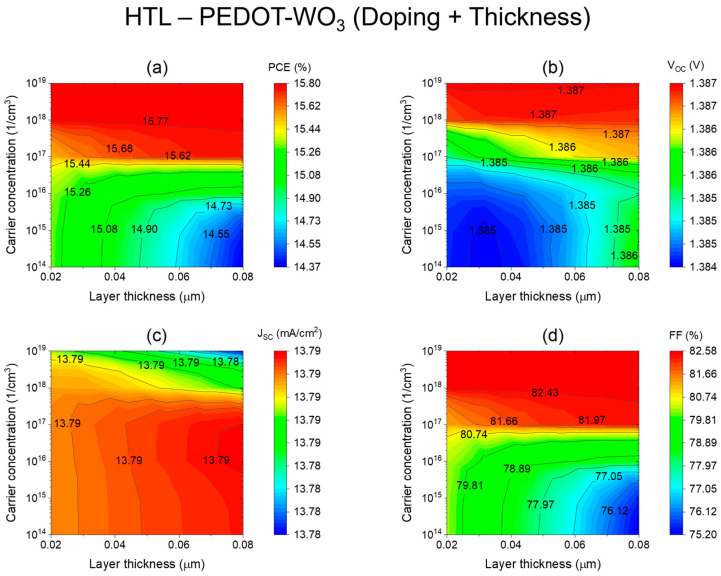
Impact of the HTL (PEDOT-WO_3_) thickness and carrier concentration on key photovoltaic parameters: (**a**) Power Conversion Efficiency (PCE). (**b**) Open-Circuit Voltage (*V_oc_*). (**c**) Short-Circuit Current Density (Jsc). (**d**) Fill Factor (FF).

The optimal ZnOS thickness and carrier concentration were determined by conducting a comprehensive correlation study that examined the influence of ZnOS thickness and carrier concentration on device performance. The doping level of ZnOS-ETL was systematically tuned across a range of 10^12^ cm^−3^ to 10^19^ cm^−3^, while the thickness was varied from 10 nm to 150 nm as shown in Figure 3. Figure 3a–d illustrates the influence of ZnOS thickness (x-axis) and doping concentration (y-axis) on photovoltaic performance parameters. The findings demonstrate that increasing the thickness of the ZnOS-ETL from 0.01 to 0.12 μm results in an increase in Jsc, ranging from 13.05 to 14.18 mA/cm^2^. Additionally, Figure 4a highlights that reducing the ETL thickness from 150 nm to 120 nm while maintaining a high doping concentration of 10^19^ cm^−3^ leads to an improvement in PCE, rising from 13.7% (Figure 4a) to 15.7% (Figure 5a). With respect to carrier concentration, both *V_oc_* and FF exhibit a substantial increase of 0.2 V and 11%, respectively.

In Figure 3c, the results clearly illustrate that increasing the thickness of the ZnOS-ETL from 0.01 to 0.15 μm leads to a noticeable increase in J_sc_, ranging from 13.05 to 14.29 mA/cm². Additionally, increasing the concentration of doping has proven to be beneficial, since it reduces ZnOS thickness while achieving an impressive PCE of approximately 15.7%, as shown in Figure 3a (the red zone color). It is likely that this enhancement is due to the significantly improved conductivity of the ETL, which resulted in a more efficient collection of charge carriers at the front electrode. As thickness is increased from 10 nm to 150 nm for a carrier concentration of 10^19^ cm^−3^, *V_oc_* and FF almost reach saturation, with average values of 1.386 V and 82.4% (Figure 3b,d). These results suggest that further increasing the thickness beyond 150 nm may not significantly impact *V_oc_* and FF. These findings indicate that the device thickness should be limited to 150 nm for maximum *V_oc_* and FF. The findings indicate that thinner ETLs can offer equivalent performance while minimizing the amount of ETL material required. As a result, this reduction in material usage can significantly lower fabrication costs, making it a cost-effective approach for enhancing photovoltaic device efficiency.

The study concludes with the following statement. An increased concentration of donor carriers in the ETL enhances the efficiency of the perovskite solar cell (PSC). As a result of enhancing the conductivity of the ETL by increasing the doping concentration in the ETL, electrons are able to flow more easily between the absorber layer and the front electrode of the device. A study conducted by Xu et al. revealed that deep energy levels are formed at heterojunction interfaces when doping concentrations in ETLs are high, thereby reducing recombination and improving the overall performance of solar cells [48].

This comprehensive study and analysis led to a successful optimization of the ZnOS-ETL thickness by maintaining a high doping level (10^19^ cm^−3^). By reducing the thickness from 150 nm to 120 nm, we were able to maintain the highest PCE. Consequently, 120 nm was identified to be the optimum thickness for ZnOS ETL, maximizing overall solar cell performance.

### 3.2. Effect of HTL Materials on PSC Photovoltaics Performance Parameters

In perovskite solar cells, HTLs play an essential role in optimizing performance. Their primary function is to efficiently extract and transport photogenerated holes from the perovskite material to the back electrode, thereby minimizing charge recombination [37,38]. Many key requirements must be met in order for a material to qualify as a successful hole transport material. To ensure efficient hole extraction, the HTL should exhibit a favorable energy level alignment with the perovskite material. Additionally, it should possess high hole mobility and excellent conductivity, enabling effective charge transportation. Moreover, the HTL should demonstrate superior photo and stability, ensuring long-term reliability. The low production costs of materials are another desirable characteristic, making them an attractive alternative. Several organic and inorganic materials have been investigated as potential hole transport materials for the MaGeI_3_ PSC structure. These include Nickel Oxide (NiO), Spiro-OMeTAD (Spiro-2,2′,7,7′-tetrakis(N,N-di-p-methoxyphenylamine)9,9′-spirobifluorene), and PEDOT (Poly(3,4-ethylenedioxythiophene)). In the initial reference structure, the HTL employed was PEDOT-WO_3_ (Poly(3,4-ethylenedioxythiophene): tungsten trioxide). Table 4 presents a comprehensive list of all parameters associated with these four HTL materials, providing a detailed overview of their characteristics and properties. Organic materials are commonly used as HTLs. They are known for their limited stability. The instability arises from organic materials’ morphological changes when exposed to thermal conditions, resulting in property changes. A promising approach to addressing HTL instability involves substituting organic materials with hybrid organic–inorganic materials such as PEDOT-WO_3_. By achieving this transition, the stability issues associated with hybrid HTL can be effectively overcome, leading to improved performance and durability of PSC. A comparison of the current–voltage (J-V) characteristics of various HTL alternatives with respect to the HTL materials used in the reference sample is shown in Figure 4.

In Figure 4a, a comprehensive representation of all band alignments at the interface between the PSC and the hole transport layer (HTL) is displayed. Photogenerated carriers are trapped by incomplete bonds at the interface of a solar cell, resulting in interface recombination loss. Interface defects are produced by lattice mismatches, grain boundaries of varying sizes, and impurities injected at the interface. This happens when the absorber and HTLs form a junction. Using different HTLs for conduction/valance band alignments allows for more interface engineering. Recombination at the interface is caused by the large valance band offset (VBO) between the HTL and absorber layer. Trap states at device interfaces cause interface recombination, resulting in a lower open circuit voltage (*V_oc_*). Figure 4a illustrates two possible interfaces between the absorber and different HTLs.

A cliff-like interface is formed when the electron affinity of the absorber is lower than the electron affinity of the HTL (-VBO) for PEDOT and Spiro-OMeTAD. The opposite effect occurs when the absorber’s electron affinity exceeds the HTL, resulting in a spike-like interface with a positive valance band offset (+VBO) for PEDOT-WO_3_ and TiO_2_, with PCE 13.59% and 11.13%, respectively. As the VBO increases, the band structure of the valence band at the interface undergoes a transition from a cliff-like structure to a spike-like structure. In Figure 4b, for hole flow towards the back contact, as VBO increases for NiO HTL, the spike height increases, resulting in an elevated resistance and reduced current. This resistance contributes to a loss in the power conversion efficiency (PCE) of the solar cell. Featuring a moderate spike height, low resistance, and high current and *V_oc_*, the interface between PSC/PEDOT-WO_3_ demonstrated the best alignment of bands, leading to extremely high performance. In Figure 4b, it is evident that the photogenerated current density for PEDOT-WO3 HTL is notably higher (approximately 11 mA/cm²) compared to NiO HTL (approximately 9 mA/cm²) with very close *V_oc_* values. As a result, the FF for PEDOT-WO3 is significantly higher, contributing to a much higher PCE (13.59%) compared to NiO HTL (11.13%). It is important to note that both FF and current density are indeed directly proportional, as confirmed by the calculated PCE values in the histograms in the inset of Figure 4b. The higher PCE of PEDOT-WO3 compared to NiO is a result of its improved FF and current density, as indicated in the histograms.

The PEDOT-WO_3_ HTL material demonstrated exceptional performance, achieving a high-power conversion efficiency of 13.59% along with a short-circuit current density of 11.3 mA/cm^2^ and a *V_oc_* of 1.41 V. This remarkable outcome can be attributed to multiple factors that contribute to improved performance. Firstly, there is a notable reduction in current leakage, which minimizes the loss of charge carriers. Additionally, there is an enhancement in hole extraction characteristics, facilitating more efficient transport of positive charges. Furthermore, there is a decrease in trap-assisted interfacial recombination, which typically hinders the overall performance.

Figure 4c shows that only when the light wavelength is below 350 nm do the four HTLs have identical quantum efficiency behavior. In the specific context of wavelengths around 688 nm in the visible light spectrum, it becomes evident that PEDOT and PEDOT-WO_3_, in comparison to other Hole Transport Layers (HTLs), showcase superior transparency, resulting in remarkably high quantum efficiency. This enhanced performance can be directly attributed to the specific bandgap energy of these materials. Indeed, both materials exhibit remarkably close bandgap energies. Specifically, PEDOT possesses an energy gap of 1.75 eV, while PEDOT-WO3 has a slightly higher energy gap of 1.8 eV. Consequently, the observed behavior of the two curves can be attributed to this shared characteristic of their bandgap energies. However, PEDOT-WO_3_ demonstrated enhanced absorption behavior at wavelengths below 680 nm compared with bare PEDOT, along with a slightly higher external quantum efficiency (EQE). Therefore, considering the observed superior absorption behavior and slightly higher EQE, the PSC structure was designed incorporating PEDOT-WO_3_ as the preferred choice for the HTL material.

Figure 5a–d illustrates the impact of PEDOT-WO3 thickness (x-axis) and doping concentration (y-axis) on the photovoltaic performance parameters. The results reveal that increasing the thickness of the Hole Transport Layer (HTL) from 20 nm to 80 nm leads to a decrease in Power Conversion Efficiency (PCE) when the HTL doping level is below 10^19^ cm^−3^. Our goal was to maintain high doping levels of 10^19^ cm^−3^ while optimizing the PEDOT-WO_3_ hole transport layer thickness. However, surpassing this doping level by increasing the concentration of donor carriers results in further reduction of PEDOT-WO_3_ thickness, with values as low as 20 nm. Interestingly, the device’s power conversion efficiency was observed to vary almost independently of the HTL layer thickness at this doping level (Figure 5a). This allowed us to achieve an impressive PCE of 15.8% at very low thicknesses. An ultra-thin PEDOT-WO3 HTL of 10^19^ cm^−3^ was determined to be the best value for the PSC structure, while an ultra-thin PEDOT-WO_3_ HTL of 20 nm was more cost-effective. These findings offer valuable insights for improving both the efficiency and cost-effectiveness of the photovoltaic device.

### 3.3. Impact of ETL and HTL Materials as a Function of Absorber Thickness

An examination of the impact of different ETLs and HTLs on PCE was performed in this numerical study. The primary parameters of the materials integrated and incorporated in this research are presented in Table 3 and Table 4. These tables provide a comprehensive overview of the crucial material characteristics that were analyzed in our investigation. This study is focusing on analyzing the performance of each ETL/HTL material in relation to the thickness of the absorber layer (MAGeI_3_), which ranges from 150 nm to 1000 nm, as shown in Figure 2.

The results of this study showed that the optimal thickness of the absorber layer is 560 nm. Furthermore, the results indicated that the choice of ETL and HTL materials can significantly affect the overall device performance. Interestingly, it was observed that the power conversion efficiency of the device exhibited an upward trend as the absorber layer thickness increased from 150 nm to 600 nm, regardless of the ETL and HTL materials utilized. This phenomenon can be attributed to the higher rate of photogenerated charge carriers, resulting from an increased light absorption within the thicker active layer. Above an absorber thickness of 600 nm, the power conversion efficiency (PCE) of the device exhibited saturation and a slight degradation. This can be attributed to an increased recombination rate between electrons and holes as the MAGeI_3_ layer becomes thicker, occurring just before the electrons and holes reach the front and back electrodes of the device.

**Figure 6:**
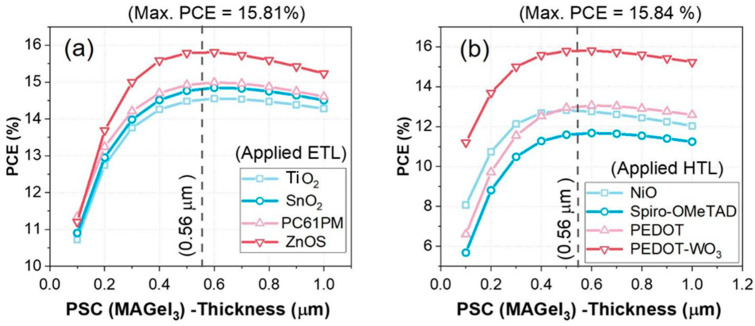
Displays the influence of active layer thickness (MAGeI_3_) ranging from 100 nm to 1000 nm on power conversion efficiency (PCE). The PCE is studied with respect to the device configuration: FTO/(120 nm) ETL/(100–1000 nm) MAGeI_3_ (absorber)/(20 nm) HTL. The impact of absorber thickness is investigated as a function of: (**a**) Variation in applied ETL materials. (**b**) Variation in applied HTL materials.

Figure 6a depicts that the ZnOS ETL material achieves the highest PCE at 15.81% at absorber thickness of 560 nm. These results strongly indicate that this specific configuration, with optimized band alignments, effectively reduces both optical and electrical losses within the device during operation under standard test conditions, as discussed in Section 2. Figure 6b clearly demonstrates that the maximum power conversion efficiency (PCE) of 15.81% is observed for the PEDOT-WO_3_ HTL material. This finding highlights the effectiveness of this material in reducing the rate of recombination at the back side of the photovoltaic (PV) device.

### 3.4. Impact of Temperature on FTO/ZnOS/MAGeI_3_/PEDOT-WO_3_/Ag Structure

**Figure 7:**
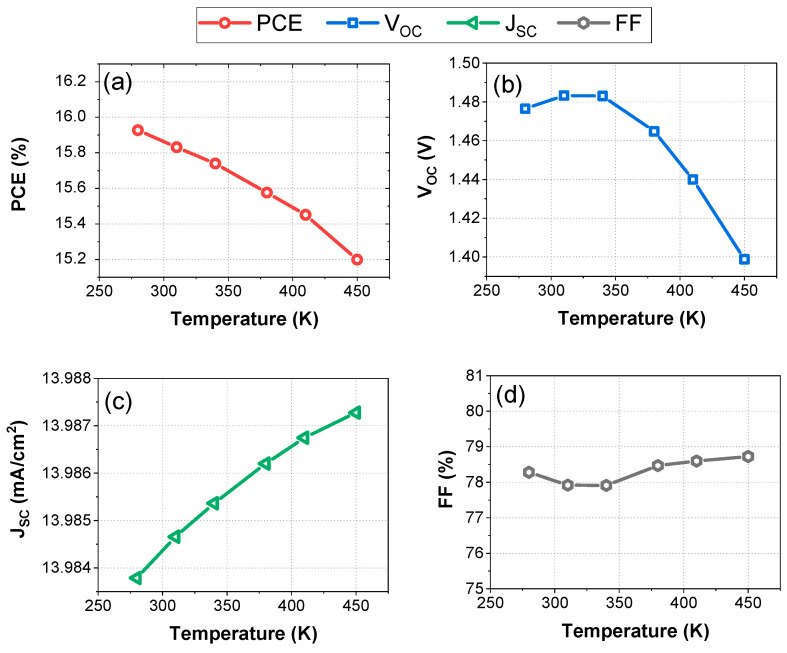
Variation of photovoltaic parameters with temperature in the range of (280 K to 450 K): (**a**) Power Conversion Efficiency (PCE). (**b**) Open-Circuit Voltage (*V_oc_)*. (**c**) Short-Circuit Current Density (Jsc). (**d**) Fill Factor (FF).

This section explores the influence of operating temperature on the FTO/ZnOS/MAGeI_3_/PEDOT-WO_3_/Ag PSC structure within the temperature range of 275 K to 450 K. Figure 7a–d illustrates the effects of temperature on important photovoltaic performance parameters, including PCE, FF, *V_oc_*, and Jsc.

Figure 5a–d illustrates the impact of PEDOT-WO_3_ thickness (x-axis) and doping concentration (y-axis) on the photovoltaic performance parameters. The results reveal that increasing the thickness of the Hole Transport Layer (HTL) from 20 nm to 80 nm leads to a decrease in Power Conversion Efficiency (PCE) when the HTL doping level is below 10^18^ cm^−3^. However, surpassing this doping level by increasing the concentration of donor carriers results in further reduction of PEDOT-WO_3_ thickness, with values as low as 20 nm. These particular thickness and doping concentration values are considered optimal for the utilized PEDOT-WO_3_ HTL in this study.

The device’s PCE exhibited a decrease of 0.7% throughout the entire temperature range, demonstrating its high stability even at elevated temperatures. Remarkably, the PCE of the device experienced only a slight decrease of 0.4% at a temperature as high as 372 K, as demonstrated in Figure 7a. In contrast, Figure 7b reveals a substantial decline in *V_oc_*, with a reduction of 0.8 V observed within the investigated temperature range. The reduction in open circuit voltage can be attributed to an increase in the reverse saturation current density (J_o_). This increase is primarily caused by two factors: a decrease in the semiconductor bandgap and an increase in the intrinsic carrier concentration (*ni*). These combined effects lead to a decrease in the open circuit voltage observed in the study [49]. The decrease in *V_oc_* as temperature increases is supported by Equation (1), which establishes a relationship between *V_oc_* and the saturation current Io [50,51].

(1) $$V_{oc}=n{\;V}_{T}ln\frac{{i_{p}+I}_{0}}{I_{0}}$$

where the symbol $i_{p}$ represents the photocurrent, $I_{0}$ denotes the saturated current, and n is the intrinsic concentration. This phenomenon results in an increase in the dark current in the PSC device. Furthermore, the temperature rise affects the material conductivity, leading to degradation in the performance of the PSC solar cell. Therefore, as the temperature increases, the band gap of the material decreases, resulting in an accelerated recombination of electrons and holes between the conduction and valence bands. These findings agree with previously reported studies on solar cells’ temperature-dependent performance [36,37]. Figure 7c illustrates that as the temperature increased, there was a significant decrease in *V_oc_*, ranging from 1.49 V to 1.4 V. However, Jsc and FF showed minimal change and exhibited a more saturated behavior, with FF values between 78% and 79% (Figure 7b,c) and a constant current density of 13.986 mA/cm^2^. Interestingly, the overall conversion efficiency of the perovskite solar cell (PSC) demonstrated good stability even at higher operating temperatures.

## 4. Conclusions

In conclusion, our study focused on investigating a lead-free MAGeI_3_ perovskite solar cell (PSC) device structure, with the primary objective of identifying the ideal combination of carrier transport materials. We specifically explored various configurations of ETLs and HTLs, focusing on a hybrid organic–inorganic perovskite solar cell as the active material. After evaluating different configurations, we found that the FTO/ZnOS/MAGeI_3_/PEDOT-WO3 configuration exhibited the highest power conversion efficiency (PCE) and proved to be the most suitable option. This optimized structure significantly reduced current leakage, increased carrier mobility, and improved electron and hole extraction efficiency, while also minimizing trap-assisted interfacial recombination. Additionally, by optimizing the active layer thickness to 560 nm, we further enhanced the solar cell performance. This work represents a significant step forward in the development of efficient, more stable, and ultra-thin perovskite solar cells, paving the way for their potential implementation in practical applications.

## Figures and Tables

**Table 1:** A list of all physical parameters applied in the simulated reference model at 300 K.

Parameters	ETL (TiO_2_)	Absorber (MAGeI_3_)	HTL (PEDOT-WO_3_)
Thickness (μm)	0.025 *	0.4 *	0.15
Bandgap (eV)	3.20	1.3	1.8
Electron affinity (eV)	3.9	3.92	3.4
Dielectric permittivity	10	10	18
CB effective DOS (cm^−3^)	2 × 10^20^	1 × 10^16^	2.2 × 10^18^
VB effective DOS (cm^−3^)	1 × 10^19^	1.8 × 10^16^	1.8 × 10^19^
Electron mobility (cm^2^/V·s)	20	16	0.045
Hole mobility (cm^2^/V·s)	10	10	0.045
Donor density N_D_ (cm^−3^)	1 × 10^19^ *	1 × 10^14^ *	0
Acceptor density N_A_ (cm^−3^)	0	1 × 10^14^ *	1.8 × 10^19^
Defect type	SA	Neutral	Neutral
Energetic distribution	Single	Gaussian	Gaussian
Defect density (cm^−3^)	1 × 10^15^	1 × 10^14^	1 × 10^15^

* Variable field.

**Table 2:** Interface defect parameters utilized in the simulation.

Parameters	HTL/MAGeI_3_ Interface	MAGeI_3_/ETL Interface
Defect type	Neutral	Single Acceptor (SA)
Capture cross section of electrons (cm^2^)	1 × 10^−18^	1 × 10^−18^
Capture cross section of holes (cm^2^)	1 × 10^−18^	1 × 10^−17^
Reference for the defect energy level E_t_	Above E_v_	Above E_v_
Energy with respect to reference (eV)	0.6	0.6
Total density (cm^−2^)	1 × 10^15^	1 × 10^14^

**Table 3:** A list of all physical parameters applied in the simulation for ETL materials at 300 K.

Parameters	Applied ETLs			
	TiO_2_	SnO_2_	PC61PM	ZnOS
Thickness (μm)	0.025 *	0.025 *	0.025 *	0.025 *
Bandgap (eV)	3.20	3.6	2.1	3.5
Electron affinity (eV)	3.9	4	4	3.6
Dielectric permittivity	10	10	18	9
CB effective DOS (cm^−3^)	2 × 10^20^	2.2× 10^18^	2.2× 10^18^	2.2× 10^18^
VB effective DOS (cm^−3^)	1 × 10^19^	1.8 × 10^19^	1.8 × 10^19^	1.8 × 10^19^
Electron mobility (cm^2^/V-s)	20	80	0.002	100
Hole mobility (cm^2^/V-s)	10	15	0.002	25
Donor density N_D_ (cm^−3^)	1 × 10^18^ *	1 × 10^18^	1 × 10^18^	1 × 10^18^ *
Acceptor density N_A_ (cm^−3^)	0	0	0	0
Defect type	SA	Neutral	Neutral	Neutral
Energetic distribution	Single	Gaussian	Gaussian	Gaussian
Defect density (cm^−3^)	1 × 10^15^	1 × 10^15^	1 × 10^15^	1 × 10^15^

* Variable field.

**Table 4:** A list of all physical parameters applied in the simulation for HTL materials at 300 K.

Parameters	Applied HTLs			
	NiO	Spiro-OMeTAD	PEDOT	PEDOT-WO_3_
Thickness (μm)	0.15	0.15	0.15	0.15 *
Bandgap (eV)	3.8	2.88	1.75	1.8
Electron affinity (eV)	1.46	2.05	3.4	3.45
Dielectric permittivity	10.7	3	3	18
CB effective DOS (cm^−3^)	2.8 × 10^18^	2.2 × 10^18^	2.2 × 10^18^	2.2 × 10^18^
VB effective DOS (cm^−3^)	1 × 10^19^	1.8 × 10^19^	1.8 × 10^19^	1.8 × 10^19^
Electron mobility (cm^2^/V-s)	12	2 × 10^−4^	0.045	0.045
Hole mobility (cm^2^/V-s)	2.8	2 × 10^−4^	0.045	0.045
Donor density N_D_ (cm^−3^)	0	0	0	0
Acceptor density N_A_ (cm^−3^)	1 × 10^18^	1 × 10^19^	1× 10^18^	1 × 10^18^ *
Defect type	Neutral	Neutral	Neutral	Neutral
Energetic distribution	Gaussian	Gaussian	Gaussian	Gaussian
Defect density (cm^−3^)	1 × 10^15^	1 × 10^15^	1 × 10^15^	1 × 10^15^

* Variable field.

## Data Availability

Data available upon request.
